# The Human Cell Atlas: making ‘cell space’ for disease

**DOI:** 10.1242/dmm.037622

**Published:** 2019-02-01

**Authors:** Chris P. Ponting

**Affiliations:** MRC Human Genetics Unit, University of Edinburgh, Crewe Road, Edinburgh EH4 2XU, UK

**Keywords:** Human Cell Atlas, Single-cell genomics, Single-cell transcriptomics

## Abstract

A single change in DNA, RNA, proteins or cellular images can be useful as a biomarker of disease onset or progression. With high-throughput molecular phenotyping of single cells, it is now conceivable that the molecular changes occurring across thousands, or tens of thousands, of individual cells could additionally be considered as a disease biomarker. Transition to a disease state would then be reflected by the shifts in cell numbers and locations across a multidimensional space that is defined by the molecular content of cells. Realising this ambition requires a robust formulation of such a multidimensional ‘cell space’*.* This is one of the goals of the recently launched Human Cell Atlas project. A second goal is to populate this ‘cell space’ with all cell types in the human body. Here, I consider the potential of the Human Cell Atlas project for improving our description and understanding of the cell-type specificity of disease.

## Abstract (precis)

**Summary:** In this Special Article, the author reflects on the potential of the Human Cell Atlas project for improving our understanding of disease.

## Introduction

Can we attribute a disease to a cell type? If so, then what molecular features of a cell type best predict a disease state? Could we pinpoint the origin of a disease both to a particular cell type and to a defined developmental time point? Answers to these questions will help to deliver on the promise of new therapies, specifically those that target disease onset or progression, by taking advantage of the distinctive features of cell types.

An obstacle that needs to be surmounted before this promise is realised is our incomplete knowledge of how to define and distinguish cell types. It is this knowledge gap that the Human Cell Atlas (Regev et al., 2017) intends to fill by defining all human cell types according to the molecules that they typically contain. This Atlas, and the information it contains, would then allow researchers to infer a cell type's abundance, physiological states, developmental trajectories and spatiotemporal locations.

The first draft of the Atlas is expected to profile 30- to 100-million cells and their matching tissues. These will be obtained from rapid autopsy or organ donors from deceased, ethnically diverse, adults (20 to 55 years old) of both sexes. In its first draft, information on most cells will be generated by 3′-tag RNA sequencing, which surveys the number of transcripts in each cell's transcriptome. Each tissue's cells will be sampled sufficiently to identify all except the most rare (<1%) of cell types. The consortium has pledged to allow unrestricted access to all the data generated, where consent agreements allow, and as soon as possible. To be fully realised, the lofty ambition of the Human Cell Atlas will need to successfully address a number of technical and analytical challenges (Box 1). Here, I assume that these all come to pass in the near future.

**Box 1. Current technical and analytical challenges in single-cell biology**

**Efficient isolation**

Some cell types are robust and remain viable following tissue digestion, cell sorting and/or suspension protocols; others are considerably more fickle and these are at risk of being under-sampled.

**Cost**

Large-scale single-cell and spatial transcriptomics or proteomics experiments are not currently routine in most laboratories owing to their high costs, although combinatorial labelling for sequencing experiments has recently reduced these considerably (Cao et al., 2017).

**Batch effects**

Technical differences will exist among experiments performed on different platforms, at different times and/or in different laboratories, and these need to be accounted for (Haghverdi et al., 2018).

**Cell-type annotation**

In general, different cell types are easily discriminated as separate clusters following the clustering of sparse single-cell transcript or epigenetic data (Prakadan et al., 2017; Mezger et al., 2018). The Human Cell Atlas Consortium, however, has not sought to define cell type*.* Rather, it believes that a robust definition will eventually emerge from empirical observation. Assignment to a type implies that a particular cell shares phenotypic and functional features with other cells of the same type. However, single-cell data, considered alone, are limited to only predicting, rather than demonstrating, cellular functionality. Consequently, independent experimental investigation of cell-type function is necessary.

**Cell-state inference**

Cells of a particular type are likely to occupy a continuum of states, owing to the cell cycle, or differentiation, or spatial location, for example (Wagner et al., 2016; Clevers et al., 2017). To assign cell state, therefore, we need to resist being categorical, and instead predict the continuous trajectories of cell-state change. When it is unclear whether these are cell states or types, groups of similar cells may best be described as (sub-) populations. Going beyond measurements of RNA abundance, the rate by which gene expression of these populations changes can be inferred from single samples (La Manno et al., 2018).

**Multi-omic data integration**

Increasingly, several different data types will be measured in the same single cell, for example RNA abundance versus spatial location or open chromatin or protein abundance. Maximising the predictive value of such multi-omic data will be a key future challenge (Packer and Trapnell, 2018).

## The ‘cell space’

**Fig. 1.**
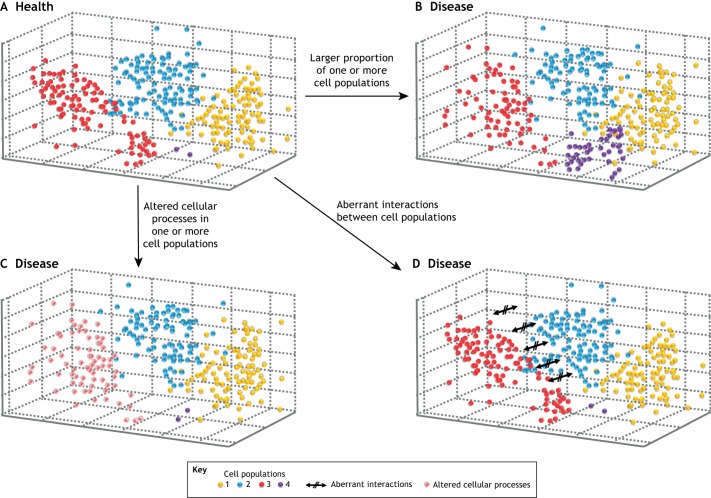
**Schematic representation of a multidimensional cell space populated by cells from healthy and disease samples.** Example healthy (A) and disease (B-D) samples are shown. Four hypothetical cell populations are shown in different colours. The location of an individual cell (represented by a sphere) in this space is determined by its molecular (e.g. RNA) content. Cells that lie in proximity in this space are expected to contain a more similar set of molecules and to be similar in cell state and/or cell type. One of the motivating hypotheses of the Human Cell Atlas is that the locations of cells from healthy samples typically differ from those of cells from disease samples.

One expected outcome of the Human Cell Atlas project is the development of a multidimensional representation, a ‘cell space’ (Trapnell, 2015; Wagner et al., 2016; Clevers et al., 2017), of the molecular similarities and differences among all known types of human cells (Fig. 1). The proximity of cells within this space implies that they are drawn from a population of similar type and state (Box 1). This population need neither to have arisen from a single developmental lineage, nor to have been spatially collocated within the original donor. This cell space would provide a reference against which other cells would be annotated with respect to type or state, simply by virtue of their collocation. Cells that project into unoccupied space could potentially represent novel cell types, although their novelty and distinctive function would require experimental verification (Box 1).

The untested, motivating hypothesis of the Human Cell Atlas is that cells from disease samples consistently project into this space differently to cells from healthy control samples (Fig. 1). Theoretically, such differences could arise from altered cell numbers (Fig. 1B) or cellular processes (Fig. 1C) for one or more cell populations. It is possible that such a space will not capture all aspects of disease pathophysiology. For example, if an RNA-based atlas does not perfectly reflect cell-cell interactions, then an RNA-defined cell space might not be able to identify the disease states that involve aberrant interactions between cell types (Fig. 1D).

In its first phase, the Human Cell Atlas project will not analyse cells from large disease-case-control cohorts (The Human Cell Atlas Consortium, 2017), so most disease mechanism studies currently lie out of scope (Rozenblatt-Rosen et al., 2017). Consequently, we expect its initial importance to stem not from the unbiased molecular definition of disease, but from the construction of a reliable multidimensional reference cell space into which any researcher can project their own single-cell data. Furthermore, the project should deliver standard experimental and analytical protocols for generating single-cell datasets and for projecting them into this common space.

Future studies will likely take advantage of the Human Cell Atlas project's experimental and analytical framework. For example, studies that robustly observe changes in cell populations across case-control cohorts could define disease status and quantify disease progression. Metrics for disease progression could be:

(1) the rate of change in the size of a disease-predictive cell subpopulation; or,
(2) the rate of change of a transcriptomic signature across one or more cell populations; or,
(3) a vector representing the shift of a cell population in multidimensional space as the disease progresses.

Disease status would then be definable by applying a threshold to the predefined disease progression metric. Conversely, drugs that significantly reverse these rates of change or this vector would represent therapeutic candidates.

These are some of the potential future benefits of the Human Cell Atlas project. Are there more near-term advantages for better understanding disease? Two recent studies of the mammalian trachea (Montoro et al., 2018; Plasschaert et al., 2018) exemplify how single-cell studies are enhancing our understanding of Mendelian disease. These studies discovered a previously unknown cell type in which the *CFTR* (cystic fibrosis transmembrane conductance regulator) gene, which is mutated in cystic fibrosis patients, is highly expressed. In this, as in future examples, the physiological consequences of gene deficiency in these rare cell types will require further detailed experimentation.

Molecular data at the single-cell resolution are also improving our understanding of the cellular response to infectious disease (Stubbington et al., 2017), injury (Arneson et al., 2018) or treatment (Vogel et al., 2016). For example, antibiotic treatment fails to substantially alter the transcriptional identities of innate lymphoid cell populations, yet it perturbs their relative proportions and their transcript abundance for several hundred genes (Gury-BenAri et al., 2016). Similar findings have been reported for mouse intestinal epithelial cells following bacterial or helminth infection (Haber et al., 2017).

Cancer makes only a minor contribution to the future plans of the Human Cell Atlas (The Human Cell Atlas Consortium, 2017). Nevertheless, the Atlas' initial datasets will be invaluable for understanding tumour heterogeneity, initiation and progression. Gene expression profiles of cell types can be used to assess the contributions of stromal cells to bulk tumour transcriptomes (Puram et al., 2017) and to infer the cell of origin in pre-cancerous conditions such as Barrett's oesophagus (Jiang et al., 2017).

Studies on neurological disease are also gaining substantially from an improved definition of cell types and states. Autism spectrum disorders, for example, have been associated with mutations in a set of genes and, because the expression of these genes is enriched in foetal and in adult neurons, in particular in inhibitory neurons, it is proposed that these are the cell types that are most profoundly dysregulated in these neurodevelopmental disorders (Wang et al., 2018).

Over the past decade, genomic studies revealed large numbers of associations between genetic variants and diverse complex traits and diseases. These DNA variants tend to lie in close chromosomal proximity to genes and functional elements that are active in the tissues that are relevant to the trait or disease (Boyle et al., 2017). It is reasonable to expect, therefore, that the survey of gene activity provided by the Human Cell Atlas will lead to a more highly resolved understanding of dysregulation at the cell population level, rather than at the heterogeneous tissue level. Nevertheless, most of the heritability of traits and complex diseases can be explained by variants that only indirectly alter the functions of core disease-related genes (Boyle et al., 2017). This implies that, even with finely resolved cell-type data, explaining the molecular mechanisms underlying complex disease genetics will remain a substantial challenge. Encouragingly, however, in some instances the expression of many genes implicated in complex diseases is restricted to particular disease-relevant cell types. This allows the candidature of these genes in disease to be further strengthened and cell processes disrupted in genetic disease to be proposed (Smillie et al., 2018).

Not all facets of disease biology will be revealed by studies of human primary cells. Diseases that originate at inaccessible developmental stages, for example, will not be informed by the Human Cell Atlas project because the samples are to be drawn predominantly from adult individuals. To address this shortcoming, organoids, which, by their nature, model organ development, could provide some insight. The Human Cell Atlas refers to a Human Organoid Project within its White Paper (The Human Cell Atlas Consortium, 2017). Nevertheless, insights from human organoids are currently restricted by their irreproducibility and by a limited understanding of the processes that guide their development (Huch et al., 2017).

For these reasons, model organisms are expected to retain their central role in elucidating the mechanisms of human disease. To facilitate comparisons with human cells, there is a need for single-cell atlases from model organisms, such as those being piloted for mouse (Han et al., 2018; Tabula Muris Consortium, 2018), *Drosophila* (Davie et al., 2018) and the planarian *Schmidtea mediterranea* (Fincher et al., 2018; Plass et al., 2018). These cell atlases would then need to be compared with the Human Cell Atlas. Comparison would take advantage of orthology relationships and could project cells of one species into the cell space of the other, or project all cell spaces into a common, unifying multidimensional space. Conceptually, this would allow a specific cell type to be annotated across multiple species, and enable researchers to rank cell types by their ‘molecular drift’, specifically the degree by which each cell type's molecular features have changed since the species' last common ancestor. These analyses thus could predict the degree by which a cell population in one species models the same population in another, a useful metric when justifying the use of models.

## Conclusions

In framing this discussion, I have avoided a previous description of the Human Cell Atlas as a periodic table for biology (Regev et al., 2017). This is because it is likely that we will eventually conceive of cell states as plastic and part of a continuum in the cell space, rather than being “the ‘atomic’ units that underlie human life” (Regev et al., 2017). Also, while the periodic table of chemical elements is useful in predicting reactions between multiple elements, we are not likely to use cell atlases to predict the details of multicellular function in the near future. The Human Cell Atlas is also different from the Human Genome Project in that it is determining a highly dynamic, rather than a highly stable, system. This means that the completion of any cell atlas will be indeterminate. Rather, the Human Cell Atlas will equip science with the concepts, experimental data and analytical tools required to measure disease states at single-molecule, single-cell, tissue, individual and population levels.

What comes next after the Human Cell Atlas project? By analogy with how the Human Genome Project was followed by surveys of large numbers of genomes, it is likely that there will be a systematic determination of cellular phenotypes across a large number of individuals, presumably when high-throughput assay costs, particularly for single-cell DNA or RNA sequencing, fall further. These cellular phenotypes could also be measured across CRISPR/Cas9 screens used to disrupt one gene in one cell for all genes and all cell types. The shift in cell space location for a cell's genotype would reflect its contribution to its cell type's specificity.

Another interesting possibility is a cell-space-association study, an extension to existing transcriptome-wide association study approaches (Gusev et al., 2016). The intent of this would be to observe which cell populations are predicted, from their genotypes, to shift in cell space coherently in disease cases compared to the shift in cell space predicted for healthy controls. In such cases, shifts that were discordant between cases and controls would provide robust evidence that a cell type causally contributes to complex disease risk. Such approaches are particularly powerful because their sole reliance on genetic variation makes environmental and other confounding effects irrelevant. Whichever direction this incipient field takes, the Human Cell Atlas project looks set to greatly aid future studies of the cellular basis of disease.
